# Feasability of Introducing a Thioether Ring in Vasopressin by *nisBTC* Co-expression in *Lactococcus lactis*

**DOI:** 10.3389/fmicb.2019.01508

**Published:** 2019-07-02

**Authors:** Qian Li, Manuel Montalban-Lopez, Oscar P. Kuipers

**Affiliations:** ^1^Department of Molecular Genetics, Groningen Biomolecular Sciences and Biotechnology Institute, University of Groningen, Groningen, Netherlands; ^2^Department of Microbiology, Faculty of Sciences, University of Granada, Granada, Spain

**Keywords:** thioether bridge, lanthipeptides, vasopressin, nisin modification machinery, mass spectrometry

## Abstract

Introducing one or more intramolecular thioether bridges in a peptide provides a promising approach to create more stable molecules with improved pharmacodynamic properties and especially to protect peptides against proteolytic degradation. Lanthipeptides are compounds that naturally possess thioether bonds in their structure. The model lanthipeptide, nisin, is produced by *Lactococcus lactis* as a core peptide fused to a leader peptide. The modification machinery responsible for nisin production, including the Ser/Thr-dehydratase NisB and the cyclase NisC, can be applied for introducing a thioether bridge into peptides fused to the nisin leader peptide, e.g., to replace a disulfide bond. Vasopressin plays a key role in water homeostasis in the human body and helps to constrict blood vessels. There are two cysteine residues in the structure of wild type vasopressin, which form a disulfide bridge in the mature peptide. Here, we show it is possible to direct the biosynthesis of vasopressin variants in such a way that the disulfide bridge is replaced by a thioether bridge using the nisin modification machinery NisBTC, albeit at low efficiency. Vasopressin mutants were fused either to the nisin leader peptide directly (Type A), after the first three rings of nisin (Type B/C), or after full nisin (Type D). The type B strategy was optimal for expression. LC-MS/MS data verified the formation of a thioether bridge, which provides proof of principle for this modification in vasopressin. This is a first step prior to the necessary increase of the production yield and further purification of these peptides to finally test their biological activity in tissue and animal models.

## Introduction

Vasopressin, a neurohypophysial hormone, was originally detected by Oliver and Schäfer (1895), who demonstrated that extracts of the pituitary gland altered blood pressure. Subsequently, vasopressin was isolated and its properties were further investigated (Vigneaud, 1952). Mammalian vasopressin was reported to be produced primarily within the hypothalamic area and then released or projected to various brain regions in response to stress, sexual stimulation, uterine dilatation, and dehydration (Donaldson and Young, 2008; Insel, 2010). Vasopressin is used in medicine for different treatments including diabetes insipidus, vasodilatory shock, and gastrointestinal bleeding. The half-life of vasopressin is 16–24 min (Czaczkes et al., 1964; Baumann and Dingman, 1976), similarly to other peptide hormones (de Vries et al., 2010).

It has been reported that cyclization of therapeutic peptides is a successful method to produce peptide analogs with improved stability and biological properties (Kluskens et al., 2009; Takatsuji et al., 2019). Cyclization can impose conformational constrains and then either enhance or compromise the interaction with their receptors to some extent (Rew et al., 2002). There are also other ways to stabilize peptides by modification, e.g., by construction of N-methyl-peptide libraries or by making backbone-cyclic peptides retaining their C-terminal peptide regions via side chain thioether covalent linkages (Kawakami et al., 2008; Takatsuji et al., 2019). However, intramolecular thioether bridge insertion has been applied on peptides like angiotensin, somatostatin, and glycoprotein D rendering molecules with higher potency (Kluskens et al., 2009). In addition, thioether bridges can render peptide analogs more stable when compared to their linear form or disulfide bond-cyclized counterparts due to the higher stability of the thioether linkage against oxidation and proteolysis compared to the disulfide bond (Tugyi et al., 2005). Therefore, we aimed at replacing a disulfide bond by a thioether to extend the half-life of vasopressin.

The chemical synthesis of thioether bridged peptides can be costly and time-consuming, whereas the biological synthesis of these peptides is often more straightforward and amenable to mutational studies (Koeller and Wong, 2001; Kluskens et al., 2005; Guzmán et al., 2007; van Heel et al., 2013; Montalbán-López et al., 2017). Lanthipeptides are ribosomally produced peptides containing lanthionine. The specific modification and secretion machinery of nisin, a paradigm lanthipeptide, produced by *Lactococcus lactis*, includes NisB, NisC, and NisT. Nisin is first produced as a fusion of a leader peptide and a core peptide, where the leader peptide is recognized by NisB, NisC, and NisT (Lubelski et al., 2008). Serine and threonine residues in the core peptide can be dehydrated by the dehydratase NisB to become dehydroalanines (Dha) or dehydrobutyrines (Dhb), respectively. Dehydrated serine or threonine residues can then be regio- and stereo-specifically coupled to a cysteine by the cyclase NisC. The modified peptide is transported out of the cell via the ABC-transporter NisT. Notably, NisB, NisC, and NisT have a relaxed substrate specificity and various peptides fused to the nisin leader peptide can be efficiently modified (Lubelski et al., 2008). Engineering of this biosynthesis machinery rendered a two-plasmid platform (Supplementary Figure S1) where the modification enzymes and transport protein are expressed from a pIL253-derivative and the engineered leader-containing peptides are encoded on a pNZ8048-derivative, both under the control of a nisin-inducible promoter (Rink et al., 2005). This enables easy manipulation and controlled expression of peptides of interest, either related to lantibiotic sequences, or unrelated ones (Lubelski et al., 2008; Montalbán-López et al., 2017). It has been successfully applied to diverse peptides attached to the nisin leader peptide such as angiotensin, diverse lantibiotics identified using genome mining, and nisin fusions to antimicrobial peptides active against Gram-negative bacteria (Kluskens et al., 2009; van Heel et al., 2016; Li et al., 2018). All these could be expressed in the culture supernatants, purified and activated upon removal of the nisin leader peptide.

Due to the interest in vasopressin and other similar peptide hormones such as oxytocin, and the feasibility of using the nisin modification machinery to replace the disulfide bridge by lanthionine, we designed several mutants that could render mature, thioether-stabilized vasopressin, replacing a Cys by a Ser in such a way that the serine residue can be dehydrated by NisB, and then coupled to the thiol group of the cysteine via the cyclase NisC to form a thioether bridge. In several constructs, the production of thioether-containing vasopressin was indeed detected and characterized. However, further optimization of the yield of the modified peptide will be required to be able to characterize the stability and bioactivity of the modified peptide.

## Materials and Methods

### Bacterial Strains, Plasmids, and Chemicals

The bacterial strains and vectors used in this work are listed in Table 1. *L. lactis* was used for expression of the modified peptides and cultured in M17 broth supplemented with 0.5% (w/v) glucose (GM17) or GM17 agar for genetic manipulation or in minimal expression medium (MEM) (Rink et al., 2005) for protein expression at 30°C.

**Table 1:** Strains and plasmids used in this work.

Strains or plasmids	Characteristics	Purpose	References
**Strains**			
*L. lactis* NZ9000	*pepN::nisRK*	Expression host and indicator strain	Kuipers et al., 1997
**Vectors**			
pNZnisA-E3	EryR, *nisA*	Nisin expression	Kuipers et al., 2004
pNZnisA leader6H	CmR, *nisA*, encoding nisin, with 6-his residues inserted behind the first methionine	Expression vector, expression of a 6-his tagged nisin	Zhou et al., 2016
pNZE3 empty	EryR	Expression vector	van Heel et al., 2013
pNZ8048	CmR	Expression vector	Kuipers et al., 1997
pIL3BTC	CmR, *nisBTC*, under the control of PnisA	Modification and transport of lantibiotics	Rink et al., 2005
pIL3EryBTC	EryR, *nisBTC*, under the control of PnisA	Modification and transport of lantibiotics	van Heel et al., 2013
pNZE-nisleader-G-VSP	EryR, G-SYFQNCPRG	Expression of vasopressin, with a glycine in front of vasopressin	This work
pNZE-nisleader-ASPRG-VSP	EryR, ASPRG-SYFQNCPRG	Expression of vasopressin, with “ASPRG” in front of vasopressin	This work
pNZE-nisleader-NG-VSP	EryR, NG-SYFQNCPRG	Expression of vasopressin, with asparagine and glycine in front of vasopressin	This work
pNZE-nisleader-MG-VSP	EryR, MG-SYFQNCPRG	Expression of vasopressin, with methionine and glycine in front of vasopressin	This work
pNZE-nisleader-WG-VSP	EryR, WG-SYFQNCPRG	Expression of vasopressin, with tryptophan and glycine in front of vasopressin	This work
pNZE-nisleader-EG-VSP	EryR, EG-SYFQNCPRG	Expression of vasopressin, with glutamic acid and glycine in front of vasopressin	This work
pNZE-nis(Δ23-34)-G-VSP	EryR, *nisA(Δ23-34)*, G-SYFQNCPRG	Expression of hybrid peptide, with a glycine in front of vasopressin	This work
pNZE-nis(Δ23-34)-ASPRG-VSP	EryR, *nisA(Δ23-34)*, G-SYFQNCPRG	Expression of hybrid peptide, with “ASPRG” in front of vasopressin	This work
pNZE-nis(Δ23-34)-NG-VSP	EryR, *nisA(Δ23-34)*, NG-SYFQNCPRG	Expression of hybrid peptide, with asparagine and glycine in front of vasopressin	This work
pNZE-nis(Δ23-34)-MG-VSP	EryR, *nisA(Δ23-34)*, MG-SYFQNCPRG	Expression of hybrid peptide, with methionine and glycine in front of vasopressin	This work
pNZE-nis(Δ23-34)-WG-VSP	EryR, *nisA(Δ23-34)*, WG-SYFQNCPRG	Expression of hybrid peptide, with tryptophan and glycine in front of vasopressin	This work
pNZE-nis(Δ23-34)-EG-VSP	EryR, *nisA(Δ23-34)*, EG-SYFQNCPRG	Expression of hybrid peptide, with glutamic acid and glycine in front of vasopressin	This work
pNZ-nis(Δ23-34)-NG-VSP	CmR, *nisA(Δ23-34)*, NG-SYFQNCPRG	Expression of hybrid peptide, with asparagine and glycine in front of vasopressin	This work
pNZ-nis(Δ23-34)-MG-VSP	CmR, *nisA(Δ23-34)*, MG-SYFQNCPRG	Expression of hybrid peptide, with methionine and glycine in front of vasopressin	This work
pNZ-nisA-NG-VSP	CmR, *nisA*, NG-SYFQNCPRG	Expression of hybrid peptide, with asparagine and glycine in front of vasopressin	This work
pNZ-nisA-MG-VSP	CmR, *nisA*, MG-SYFQNCPRG	Expression of hybrid peptide, with methionine and glycine in front of vasopressin	This work

*CmR, chloramphenicol resistance; EryR, erythromycin resistance.*

Chloramphenicol and/or erythromycin were used at 5 μg/mL when appropriate.

The plasmids pNZnisA-E3 (Kuipers et al., 2004) and pNZnisA leader6H were used for the expression of nisin and as a template for the construction of the designed hybrid peptides. Plasmids pIL3BTC (Rink et al., 2005) and pIL3EryBTC (van Heel et al., 2013) encoding the nisin modification machinery were used to produce and modify the fusion peptides.

Commercial vasopressin [(Arg^8^)-Vasopressin acetate salt] was ordered from Sigma-Aldrich as a control. Endoproteinase Glu-C from *Staphylococcus aureus* V8 (V8), cyanogen bromide (CNBr), hydroxylamine (NH_2_OH), 3-Bromo-3-methyl-2-(2-nitrophenylthio)-3H-indole (BNPS-Skatole), and trypsin were ordered from Sigma-Aldrich as cleavage reagents. Ammonium acetate (CH_3_COONH_4_), formic acid, guanidine hydrochloride (Gdn-HCl), and lactic acid were ordered from Sigma-Aldrich. All the chemical reagents are of analytic purity.

### Construction of Expression Vectors

**FIGURE 1:**
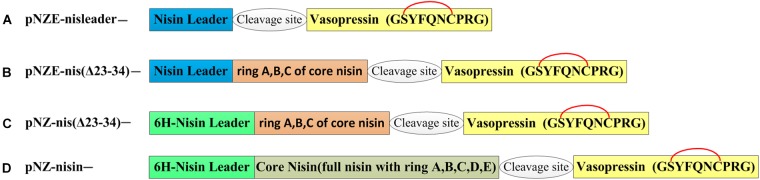
Organization of hybrid peptides. The yellow box corresponds to the mutant vasopressin of which the first cysteine was changed to serine to form a thioether bridge between dehydrated serine and cysteine with the nisin machinery. A glycine is in the front of the vasopressin sequence after cleavage of the nisin moiety. The putative thioether bridge is labeled in red. 6H-leader means that there are six histidine at the N-terminus of the nisin leader sequence. **(A)** pNZE-derived vectors harboring nisin leader and vasopressin hybrid genes. **(B)** pNZE-derived vectors harboring **(A–C)** rings of nisin and vasopressin hybrid genes. **(C)** pNZnisA leader6H-derived vectors harboring **(A–C)** rings of nisin and vasopressin hybrid genes. **(D)** pNZnisA leader6H-derived vectors harboring nisin and vasopressin hybrid genes.

The vectors harboring different coding sequences for nisin and vasopressin hybrid peptides, listed in Table 1, were obtained by round PCR as described previously (Li et al., 2018). Fast digest restriction enzymes and ligase were supplied by Thermo-Fischer and used according to the manufacturer’s instructions. In order to introduce a thioether ring into vasopressin, the amino acid sequence was changed from “CYFQNCPRG” into “SYFQNCPRG” (Figure 1). The primers (Supplementary Table S1) were designed according to the new sequences and insert the vasopressin sequence between the nisin part and the restriction site *Hind*III. Notably, different protease/chemical cleavage sites were included in front of the vasopressin part (Table 2). Each pair of primers contained a part annealing with the template vector pNZnisA-E3 or pNZnisA leader6H, and a part encoding vasopressin. First, we made pNZ-nisA-NG-VSP and pNZ-nisA-MG-VSP, which include the whole sequence of nisin A. These vectors served as a template to construct all the other vectors containing the same cleavage sites, which shared the same forward primers with each other, and a variable reverse primer depending on the length of the nisin part to be fused to [e.g., primer named “EG-VSP-fwd” can be used as forward primers for both pNZE-nis(Δ23-34)-EG-VSP and pNZE-nisleader-MG-VSP]. This round PCR strategy served for both pNZ-nisA-E3 and pNZnisA leader6H derivatives. The constructions of expression plasmids can be divided into three different types, according to the part of nisin harbored in the plasmids (Figure 1). The part on nisin preceding the vasopressin was expected to increase the processivity of the modification enzymes giving them a “head” start and thus enhance good modification of the vasopressin part too.

**Table 2:** Cleavage methods.

Preferred sequence cleaved	Sample conditions used	References
**Protease cleavage**		
-K-↓-G-Vsp	Trypsin in 5 mM CaCl_2_ and 50 mM Tris, pH 6.0, 37°C, overnight	Mu et al., 2015
-ASPR-↓-G-Vsp	NisP in 50 mM CH_3_COONH_4_, pH 6.0, 37°C, overnight	Li et al., 2018
-E-↓-G-Vsp	a ratio of 1:20 (w/w) of V8 to substrate, 50 mM NH_4_HCO_3_, pH 7.8, 37°C, overnight	Toews et al., 2010
**Chemical cleavage**		
-N-↓-G-Vsp	1 M NH_2_OH+6 M Gdn-HCl, pH 9.0, 45°C, 2∼3 h	Park et al., 2001; Hwang et al., 2014
-M-↓-G-Vsp	100 mM CNBr in 70% formic acid, room temperature, overnight	Kaiser and Metzka, 1999; Hwang et al., 2014
-W-↓-G-Vsp	10 mM BNPS-skatole, 80% acetic acid, 47°C, 1 h	Rahali and Gueguen, 1999; Hwang et al., 2014

After amplification, the cleaned-up PCR products were digested using *Dpn*I to remove the template and ligated over-night. The ligation product was desalted and transformed into *L. lactis* NZ9000 according to standard procedures (Holo and Nes, 1995), isolated, extracted using a commercial plasmids extraction kit (Macherey-Nagel), and the integrity of the sequence was verified by DNA sequencing (Macrogen).

### Protein Expression and Purification

Each vector containing the mutant structural gene, either derived from pNZnisA-E3, or pNZnisA leader6H, was transformed into NZ9000 (pIL3BTC) or NZ9000 (pIL3EryBTC), respectively. Cells were first cultured overnight in GM17 medium with 5 μg/mL chloramphenicol and 5 μg/mL erythromycin and transferred into MEM medium (Rink et al., 2005) at a final concentration of 2%. Nisin was added as inducer for expression (Kuipers et al., 1995; Li et al., 2018). 5 ng/mL nisin was added twice, first at the beginning of the inoculation and then at the time when the culture reached an OD (600 nm) of 0.4–0.6. Thus, after the second addition of nisin, the final concentration of nisin was 10 ng/mL, still below the MIC value, thus subinhibitory.

Cells were harvested 3 h after the second induction by centrifugation at 4°C for 20 min at 6500 rpm and the supernatant was filtered and kept for the isolation of fusion peptides. In order to detect the designed peptides rapidly, a small volume of culture supernatant was used for precipitation using trichloroacetic acid (TCA) according to Sambrook and Russell (2001). The concentrated secreted fusion peptides were analyzed on a 16% Tricine SDS-PAGE gel (Schägger and von Jagow, 1987; Schägger, 2006) and stained with Coomassie blue (Fermentas). Precipitation experiments were performed at least in triplicate.

Alternatively, when higher amounts of peptides were required, a larger volume (≥1 L) of culture was inoculated, centrifuged, and concentrated by fast flow cationic exchange chromatography and gel filtration. The cell-free supernatant was mixed at a ratio 1:1 with a 100 mM lactic acid solution and applied to a 5 mL HiTrap SP-Sepharose (GE Healthcare) column previously equilibrated with 50 mM lactic acid pH 4.0. Bound peptides were washed with 50 mM lactic acid solution pH 4.0 and eluted with 50 mM lactic acid, 1 M NaCl pH 4.0 (Lubelski et al., 2009). Subsequently, a PD-10 desalting column (GE Healthcare) was used to desalt the samples following the manufacturer’s instructions. The desalted samples were freeze-dried afterward.

The peptides were purified to homogeneity by reversed-phase high performance liquid chromatography (RP-HPLC). Solvents used for RP-HPLC were solvent A (0.1% TFA in MilliQ water) and solvent B (0.1% TFA in acetonitrile). The fusion peptides were purified with a C12 column [4 μm proteo 90 Å column, 250^∗^ 4.6 mm (Phenomenex)], as indicated elsewhere (Zhou et al., 2015). Meanwhile, the peptides after digestion were purified with a C18 column [3.6 μm Aeris peptide 100 Å column, 250^∗^ 4.6 mm (Phenomenex)]. Following a 10 min washing step with 5% solvent B, a gradient of 15–30% of solvent B over 30 min was executed at a flow rate of 1 mL/min. Peptides were detected measuring the absorbance at 226 nm. The fractions were collected and analyzed by mass spectrometry (van Heel et al., 2013). The active and pure fraction was lyophilized and stored as a powder until further use.

### Cleavage of Fusion Peptides

The fusion peptides were treated with the protease NisP, trypsin or endoprotease Glu-C, or site-specific chemical reagents to remove the nisin part. NisP was purified by affinity chromatography using a Ni-NTA fast flow resin (Qiagen) as described elsewhere (Montalbán-López et al., 2018). The chemical reagents needed harsher conditions (e.g., CNBr in 70% formic acid) than the cited proteases. The cleavage methods and conditions used are listed in Table 2.

### MALDI-TOF Mass Spectrometry and Liquid Chromatography-Tandem Mass Spectrometry (LC-MS/MS)

A total of 1 μL TCA-precipitated sample or HPLC-purified fraction was loaded on the target and dried. Washing with Milli-Q water was needed if the sample was a TCA precipitation experiment. Subsequently, 1 μL of matrix solution was spotted on the washed sample. The matrix solution consisted of 5 mg/mL α-cyano-4-hydroxycinnamic acid dissolved in 50% (v/v) acetonitrile and 0.1% (v/v) trifluoroacetic acid. A Voyager DE PRO matrix-assisted laser desorption ionization time-of-flight (MALDI-TOF) mass spectrometer (Applied Biosystems) was used to obtain mass spectra using conditions previously established (van Heel et al., 2013). Data were analyzed with “Data Explorer” software version 4.0.0.0 (Applied Biosystems).

After digestion with either one of the chemicals (CNBr, NH_2_OH, and BNPS-Skatole) or proteases (NisP, trypsin, and V8) the proteolytic mix was applied to an Ultimate 3000 nano-LC-MS/MS system (Dionex) in line connected to an LTQ Orbitrap XL mass spectrometer (Thermo Fisher Scientific) as previously reported (Mu et al., 2015). The peptides were monitored by MS through 100–2000 m/z, and its molecular weight determined (Mu et al., 2015).

## Results

### Design and Expression of Fusion Peptides

**FIGURE 2:**
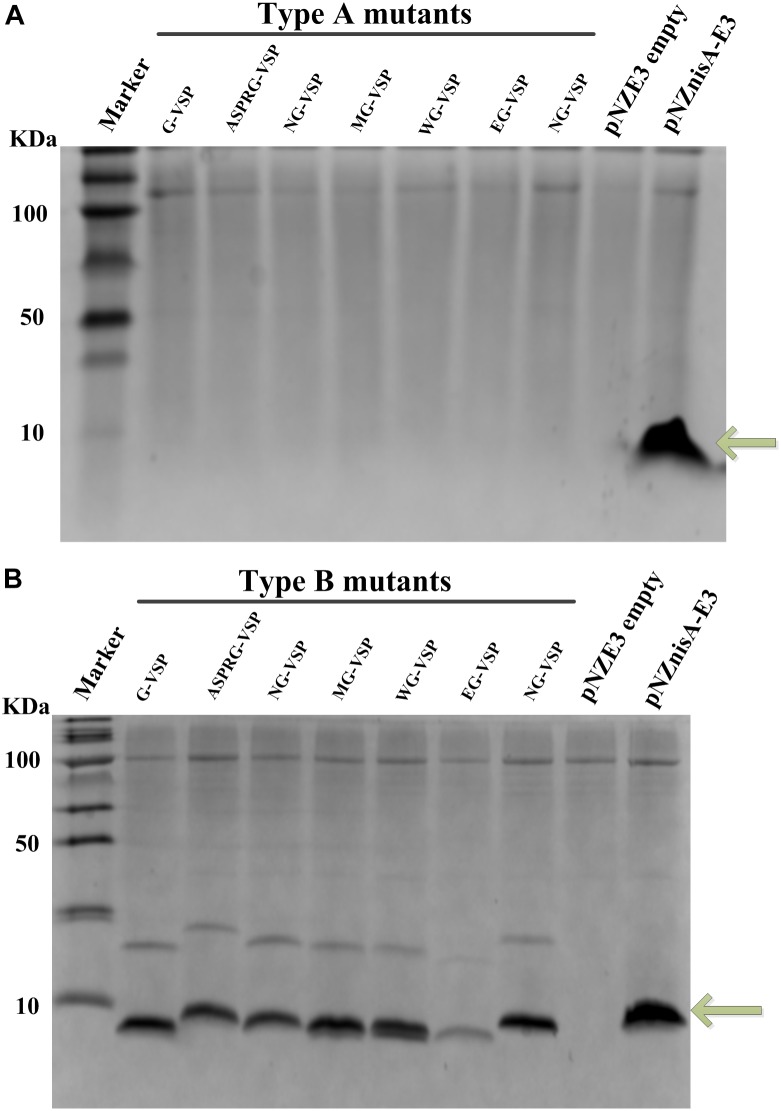
Production of vasopressin-nisin hybrids monitored by coomassie-blue stained Tricine SDS-PAGE. TCA precipitated supernatants of *L. lactis* NZ9000 (pIL3BTC, pNZE-nisin derivative) or NZ9000 (pIL3EryBTC, pNZ-nisin derivative) were loaded in each well. Marker, molecular weight marker; Wild-type nisin band produced using the same vector system as a positive control is marked by an arrow. **(A)** Type A vasopressin mutants fused to the nisin leader peptide (i.e., pNZE-nisleader-VSP mutants), positive control, NZ9000 (pNZnisA-E3, pIL3BTC); negative control, NZ9000 (pNZE3 empty, pIL3BTC). **(B)** Type B vasopressin mutants fused to rings ABC of nisin [i.e., pNZE-nis(Δ23-34)-VSP, positive control, NZ9000 (pNZnisA-E3, pIL3BTC); negative control, NZ9000 (pNZE3 empty, pIL3BTC)]. Upper light bands are most probably dimers by intermolecular disulfide formation, of those molecules where a thioether was not formed.

The sequence of wild-type vasopressin “CYFQNCPRG-NH_2_” is constrained by a disulfide bridge between the two Cys residues. In order to make this peptide amenable for the nisin modification machinery, the first Cys was mutated into Ser, generating the sequence “SYFQNCPRG.” This vasopressin mutated sequence was fused at the end of either full nisin or truncations thereof (Figure 1), inserting in each case a cleavage site for several different peptidases and chemical reagents that can facilitate the release of this hormone. Since the lanthionine ring can impose a structural constraint that limits the cleavage rate, an extra Gly residue was placed in all cases in front of the SYFQNCPRG sequence. Thus, in type A mutants (Figure 1A), the fusion was done directly to the leader peptide of nisin, whereas in type B the first rings of nisin were still present. Similarly, in type C mutants rings ABC of nisin were present, but an additional 6xHis in the leader peptide had been inserted for easier purification. Last, type D mutants consisted of vasopressin fused to full length nisin and with a His-tag in the leader. Thus, nisin and the fusion peptides containing a nisin moiety and vasopressin were produced by the nisin inducible production system previously described, which consists of NZ9000 (pIL3BTC, pNZE-nisin derivative) or NZ9000 (pIL3EryBTC, pNZ-leader6H-nisin derivative) (Kuipers et al., 2004; Rink et al., 2005; van Heel et al., 2013). In the latter case, the presence of a polyhistidine tag in the leader peptide enables purification by IMAC as well. The production levels were monitored by TCA precipitation of the supernatants and the peptides were detected by tricine SDS-PAGE. As shown in Figure 2, the production levels of the mutants obtained from the four different strategies varied greatly from each other and this mainly depended on the nisin part harbored in the vectors and the presence of the his-tag. Prepeptides formed by derivatives of pNZnisA leader6H (Type C/D) contained a his-tag, while type A and type B derivatives did not. The pNZE-nisin derivatives containing rings ABC of nisin (Type B) showed a higher production level, in a range similar to wild-type nisin produced using the same system. However, there were no detectable bands on the tricine SDS-PAGE gels of the TCA samples from the pNZE-nisin derivatives containing only the nisin leader peptide (Type A). Four pNZ-nisin derivatives with either ring A, B, and C of nisin or full nisin (Type C/D) were also checked (data not shown). pNZ-nis(Δ23-34)-NG-VSP showed a quite low production level and weak bands on the tricine SDS-PAGE gel and the production of the other three variants [pNZ-nis(Δ23-34)-MG-VSP, pNZ-nisA-NG-VSP, and pNZ-nisA-MG-VSP] could not be detected by tricine SDS-PAGE. The differences in the production levels among type A, B, and C peptides might be due either to the His-tags in the nisin leader peptide, or the increased lengths of the peptide sequences or their effects on the processivity of the modifying enzymes. The strains containing the plasmids pIL3BTC and pNZE-nis(Δ23-34)-VSP showed the best production levels. So, only type B variants were further purified and analyzed. We have found that most of the peptides were secreted out of the cell and very limited amount of peptides were present in the intracellular fraction.

### Characterization of Purified Fusion Peptides

All the type B fusion peptides were further purified via fast flow cationic exchange chromatography at a larger scale. The mass of the peptide variants before nisin part removal was determined via MALDI-TOF to assess the modification extent of the hybrid peptides, considering that a dehydration results in a mass difference of −18 Da. The results of mass analyses are listed in Table 3 and Supplementary Figure S2. For all the type B peptides, the fully dehydrated hybrid peptides were obtained in addition to partly dehydrated peptides, and we observed that the different cleavage sites (amino acids between the nisin part and vasopressin sequence) made no difference with regard to the dehydration extent of peptides. This means that, although there are molecules in which not all the serine and threonine residues in the core peptide are dehydrated, there is a large proportion of the peptide in which all the serine and threonine residues are dehydrated, including the serine present in the engineered vasopressin part. This dehydration in vasopressin enables the formation of a thioether bond with the cysteine residue present. However, to confirm the formation of the thioether rings, additional experiments such as MS/MS (see below) are needed since thioether formation results in no mass change.

**Table 3:** MS analysis and yields of type B peptides before and after nisin part removal considering the highest dehydration number possible.

Name	Predicted mass (Da)	Measured mass (Da)	Observed dehydrations/total possible dehydrations	Yield (μg/L)
**Full length type B fusion peptides**				
pNZE-nis(Δ23-34)-G-VSP	5562.71	5560.97	6/6	660
pNZE-nis(Δ23-34)-ASPRG-VSP	5955.94	5955.60	7/7	570
pNZE- nis(Δ23-34)-EG-VSP	5691.76	5691.56	6/6	760
pNZE- nis(Δ23-34)-NG-VSP	5676.75	5676.39	6/6	530
pNZE- nis(Δ23-34)-MG-VSP	5693.75	5693.35	6/6	620
pNZE- nis(Δ23-34)-WG-VSP	5748.79	5748.39	6/6	610
**Vasopressin released after cleavage of type B peptides**				
pNZE-nis(Δ23-34)-G-VSP	1110.21	1112.68	1/1	30
pNZE-nis(Δ23-34)-ASPRG-VSP	1110.21	1111.89	1/1	10
pNZE- nis(Δ23-34)-EG-VSP	1110.21	1111.63	1/1	100
pNZE- nis(Δ23-34)-NG-VSP	1110.21	1111.44	1/1	38
pNZE- nis(Δ23-34)-MG-VSP	1110.21	1110.91	1/1	54
pNZE- nis(Δ23-34)-WG-VSP	1110.21	1111.87	1/1	36

### Cleavage and Characterization of Cleaved Peptides

Fusion peptides harboring A, B, and C rings of nisin were cleaved off according to the specific cleavage site (Table 2). The masses of cleaved vasopressin were determined by MALDI-TOF and MS/MS. The results of MALDI-TOF are listed in Table 3 and Supplementary Figure S2.

After removal of the nisin moiety, vasopressin with the sequence “GSYFQNCPRG” could be obtained from all of the peptides. For all the structures, after digestion, both dehydrated and non-dehydrated vasopressin were detected, indicating partial dehydration and limited cyclization. Although MALDI is not a quantitative technique, for the peptides pNZE-nis(Δ23-34)-ASPRG-VSP, pNZE-nis(Δ23-34)-MG-VSP, and pNZE-nis(Δ23-34)-EG-VSP, the majority of the vasopressin released corresponded to dehydrated peptide, whereas in the remaining cases the peak corresponding to dehydrated VSP was too low. The chemical utilized to cleave between “M” and “G” is CNBr. CNBr is a toxic pseudohalogen (Kohn and Wilchek, 1984), thus, for large scale purification, preferably pNZE-nis(Δ23-34)-ASPRG-VSP, pNZE-nis(Δ23-34)-EG-VSP were used (could be cleaved by NisP or V8, respectively) and analyzed by HPLC and LC-MS/MS analysis.

**FIGURE 3:**
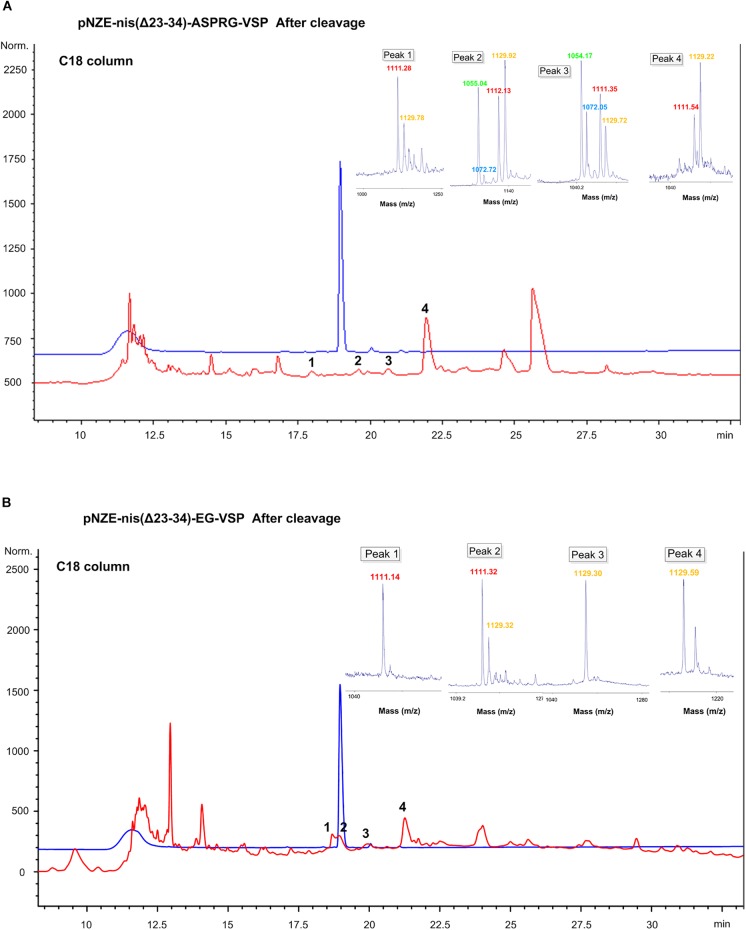
HPLC chromatograms and MALDI-TOF results of peaks collected after enzymatic cleavage of the vasopressin mutants. **(A)** pNZE-nis(Δ23-34)-ASPRG-VSP separated using a C18 column. **(B)** pNZE-nis(Δ23-34)-EG-VSP separated using C18 column. Commercial vasopressin was used as a standard and is depicted in blue line whereas the red chromatogram corresponds to the cleaved nisin-vasopressin hybrid. The mass of the peaks corresponding to vasopressin is attached to the chromatogram. For the mass spectra of collected peaks, different color correspond to different mass. Red,∼1110 Da, corresponds to the sequence of “GDhaYFQNCPRG”; yellow,∼1129 Da, corresponds to the sequence of “GSYFQNCPRG”; green,∼1054 Da, corresponds to the sequence of “GDhaYFQNCPR” with dehydrated serine; blue,∼1072 Da, correspond to the sequence of “GSYFQNCPR.” Dha, dehydroalanine (i.e., dehydrated serine).

Cleaved products of pNZE-nis(Δ23-34)-ASPRG-VSP and pNZE-nis(Δ23-34)-EG-VSP were loaded on an analytical C18 column for better separation, and purified via HPLC. Commercial vasopressin (with the sequence “CYFQNCPRG”) was used as a control (Figure 3). The commercial vasopressin and engineered vasopressin can be eluted at around 22% of solvent B.

Firstly, we analyzed NisP-treated pNZE-nis(Δ23-34)-ASPRG-VSP. The cleavage site of NisP is after proline-arginine, similarly, to the last part of vasopressin, so the last amino acid (glycine) of the vasopressin part might also be cut off. Thus, different vasopressin parts after digestion with or without the C-terminal glycine could be detected as shown by mass spectrometry. After HPLC separation, the peaks still contained a residual part of not fully dehydrated vasopressin (Figure 3A) with or without the last glycine. The injected amount of commercial vasopressin was 6 μg, and thus, we can estimate by comparison of the peak areas that the amounts of peak 1, 2, 3, and 4 were 0.28, 0.23, 0.25, and 2.3 μg, respectively. The yield of the dehydrated vasopressin from the original culture was around 10 μg/L, which was extremely low.

For pNZE-nis(Δ23-34)-EG-VSP, when a C18 column was used, four peaks were collected (Figure 3B) and most of them were non-dehydrated forms, except peak 1, which proved to be a pure dehydrated vasopressin part (Figure 3B). The amount of peptide from peak 1 was around 0.8 μg, and the yield from the original culture was around 100 μg/L, which was still not enough for further tests including stability, pharmacokinetic/pharmacodynamic tests, and animal tests.

### LC-MS/MS Fragments Analysis

**FIGURE 4:**
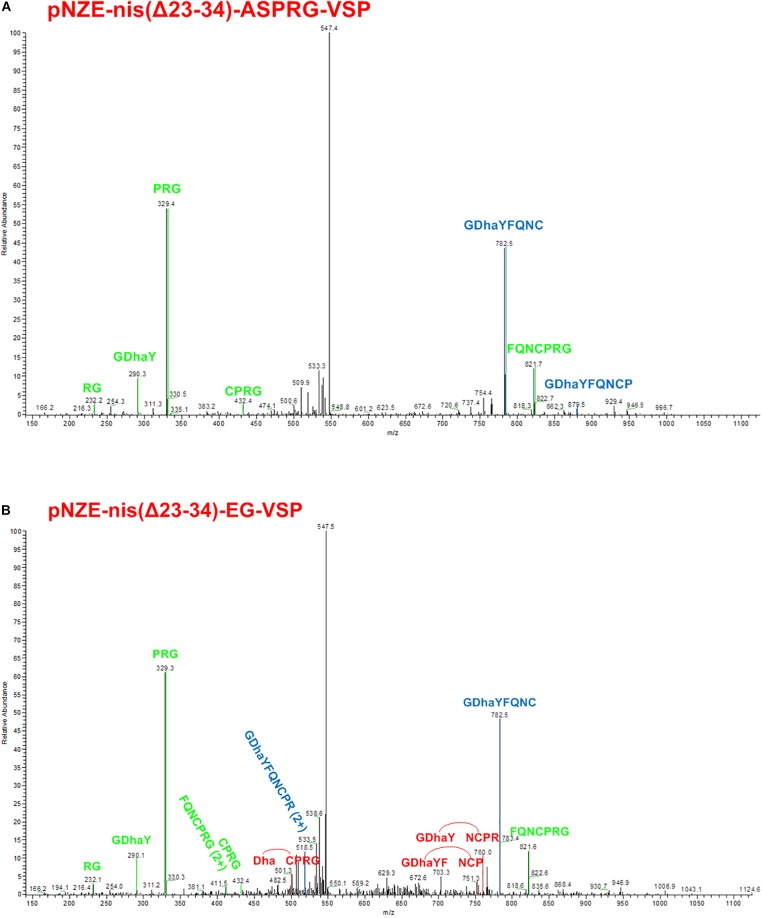
MS/MS results of characteristic fragments. **(A)** MS/MS fragments of 7 characteristic fragments labeled as green (correspond to linear vasopressin) or blue (correspond to the fragment can be either linear vasopressin or cyclized vasopressin). **(B)** MS/MS fragments of 11 characteristic fragments labeled as green (correspond to linear vasopressin), red (cyclized vasopressin), or blue (correspond to the fragment can be either linear vasopressin or cyclized vasopressin). Dha, dehydroalanine; red line between Dha and cysteine indicates a thioether bridge.

Previous MALDI-TOF results clarified the dehydration of serine in the vasopressin part in pNZE-nis(Δ23-34)-ASPRG-VSP and pNZE-nis(Δ23-34)-EG-VSP. However, more tests were needed to check if the thioether bridges were formed since there is no mass difference derived from the thioether formation. Therefore, we repeated the digestion of the peptides and the mix was subjected to LC/MS setting an appropriate mass window for the ion trap according to previous experiments that showed the double charged ion as the most abundant species in these conditions (data not shown). Vasopressin parts of both pNZE-nis(Δ23-34)-ASPRG-VSP and pNZE-nis(Δ23-34)-EG-VSP were first detected with LC/MS and then further isolated for MS/MS fragmentation (Figure 4). In this experimental setup, the chances to get fragmentation at the level of the peptide bond are higher than those to break the thioether bond. Therefore, if a lanthionine ring (i.e., thioether) is formed, fragments connecting the N- and the C-terminus of vasopressin but lacking amino acids encompassed in the lanthionine ring could be detected. Dehydrated vasopressin, with the m/z = 555.74 for the double charged ion, could be observed with a RT around 8 min [RT = 8.28 for pNZE-nis(Δ23-34)-ASPRG-VSP and RT = 8.26 for pNZE-nis(Δ23-34)-EG-VSP] and further fragmented (Supplementary Figure S3). According to the LC-MS/MS results, a fragment containing Dha can be found in both samples. Remarkably, peaks consistent with three specific fragments (DhaCPRG, GDhaYFNCP, and GDhaYNCPR) were detected in the vasopressin part of pNZE-nis(Δ23-34)-EG-VSP. If we assume that the thioether ring is formed, there would be both a thioether bridge connecting Cys and Dha and peptide bonds linking the amino acids between Dha and cysteine (Figure 1). During the fragmentation process in MS/MS determination, either of the peptide bonds can be broken, and the three specific fragments depicted above can only occur if the peptide bonds are broken and the more stable thioether bridge remained (Figure 4B). In this case, one or more amino acids between Dha and cysteine which are connected via peptide bonds might be lost, while the thioether bridge can still connect Dha, and cysteine with the Dha and cysteine bound to the other amino acids left via peptide bonds.

Thus, we can conclude that, for the fusion peptides containing the ABC rings of nisin, not all the vasopressin moieties were dehydrated. In addition, the dehydrated peptide can be either linear or cyclic with a thioether bridge. Specific fragments were found in the vasopressin part of pNZE-nis(Δ23-34)-EG-VSP, which showed the formation of a thioether ring in the peptide. Thus, we conclude that at least part of the molecules have the expected length and contain a thioether bridge.

## Discussion

It has been reported that rapid breakdown has limited the efficacy of many therapeutic peptides in complex biological fluids (de Vries et al., 2010). Cyclization can impose conformational constrains and then provide protection against proteolytic degradation as well as stabilize the conformation suitable for receptor binding (Bosma et al., 2019). Notably, a thioether link is more stable than a disulfide bond against hydrolytic decomposition, oxidation, or human serum (Tugyi et al., 2005; Rink et al., 2007). Biosynthesis would ideally allow to rapidly obtain different variants to discover improved analogs of vasopressin. Due to the interest of vasopressin and related peptide hormones (e.g., oxytocin) and the stabilizing effect of cyclization, in our work, a thioether bridge was introduced into vasopressin using the nisin modification machinery in order to create improved and more stable vasopressin variants.

Engineering of vasopressin should take into account the design and working principles of the nisin modification machinery. It has been described that, in spite of its promiscuity, NisBTC cannot equally well modify any peptide sequence fused to the nisin leader peptide (Kuipers et al., 2008) and that it can more efficiently modify peptides attached to the first rings of nisin (van Heel et al., 2016; Li et al., 2018; Schmitt et al., 2019). NisT is a broad substrate transporter that can effectively export lantibiotics but also unmodified peptides of variable length (Kuipers et al., 2004; Bosma et al., 2011). Since NisB and NisC modify from N- to C-terminus (Lubelski et al., 2009) minimally the first cysteine in the sequence of vasopressin needs to be replaced by serine to provide a dehydratable residue for NisB and thereby a substrate for a thiol attack from the remaining cysteine that renders the thioether bond. The peptide sequence surrounding the dehydratable residue is also key for modification and residues present in the cleavage site play a role. Moreover, since the presence of unusual amino acids such as Dha and the formation of the thioether bond between Dha and Cys in vasopressin could prevent cleavage, an extra Gly was always placed between the engineered cleavage site and the vasopressin sequence. The presence of N-terminal amino acids or other chemical substituents in vasopressin sequence has rendered active molecules such as terlipressin, with three glycines in this position, which is marketed for clinical use (Dobre et al., 2011; Wang et al., 2018). Thus, the presence of glycine or other amino acids at this position is tolerated in terms of activity and enables easier cleavage.

Taking into account the requirements of the nisin modification machinery exposed above we designed different groups of peptides in order to achieve the highest peptide yield. Since intracellular peptides are always incompletely modified and prone to be degraded, we only focused on the extracellular fraction. The type B peptides, with ABC rings of nisin, followed by Gly-vasopressin were proven to be optimal for production and independently of the cleavage sequence (Figure 2 and Table 3). This is in line with previous data indicating a better modification of non-cognate peptides when the N-terminus of wild-type nisin is present due to better binding to NisB (Mavaro et al., 2011; van Heel et al., 2016). Due to the higher yields and the easier and safer cleavage, the nisin part was removed with appropriate proteases (V8 or NisP) from pNZE-nis(Δ23-34)-ASPRG-VSP and pNZE-nis(Δ23-34)-EG-VSP.

After cleavage, most of the products were still a mixture of non-dehydrated and dehydrated vasopressin parts either cyclized or not, indicating that the sequence of vasopressin is not optimal for NisB and NisC. NisBC naturally render a mix of differently dehydrated peptides during nisin production and particularly when non-cognate peptides are used (Lubelski et al., 2008; van Heel et al., 2016) and that is also the case in these vasopressin mutants. It has been reported that some medically relevant non-lantibiotic peptides, including angiotensin, erythropoietin mimetic and enkephalin, were not fully dehydrated in the presence of NisB (Kluskens et al., 2005). Moreover, NisC was found to show preference and exclusion for particular amino acids at the flanking position of cysteine (Rink et al., 2007). Nevertheless, we could obtain a peptide from the strain harboring pNZE-nis(Δ23-34)-EG-VSP (Figure 3) whose mass was consistent with a thioether stabilized vasopressin albeit at very low concentration (100 μg/L). The tolerance to mutations and natural heterogeneity in other lantibiotic biosynthesis clusters in literature similarly, shows that, although the enzymes produce one main product, partly modified peptides still appear. The modification of C-terminal tails added to a truncated lacticin 481 variant induced impaired dehydration by the enzyme LctM (Zhang and van der Donk, 2007). Even ProcM, which can naturally modify up to 29 lantibiotics generates byproducts that are not fully modified (Li et al., 2010). Moreover, the alteration of the core peptide induces wrong modification patterns or leaves free cysteines as observed with NukM (Shioya et al., 2010). In fact, the sequence of the core peptide (and not only the leader peptide) has an enormous influence in the modification pattern observed with type II lantibiotic modification enzymes (Thibodeaux et al., 2014; Tang et al., 2016). The analysis of a large library of lantibiotic mutants that kept the first two rings of nisin/gallidermin also demonstrated that although NisBTC is a very suitable screening system, when they face non-cognate peptides heterogeneity and low production constitute a burden for high yield production (Schmitt et al., 2019).

MS/MS experiments unambiguously show three characteristic fragments after cleavage that prove the presence of the thioether Surprisingly, the increased length in the hinge region of nisin that is imposed by the ASPR cleavage sequence in pNZE-nis(Δ23-34)-ASPR-VSP construct allows Dha formation in engineered vasopressin but apparently reduces the cyclization efficiency by NisC since no specific fragment consistent with thioether formation was detected (Figure 4A).

Our work reports a biological way to introduce a thioether bridge in vasopressin, albeit at low efficiency, and yield. An improvement of the production yield of the peptide is essential and thus this system still requires further improvement (more efficient dehydration and cyclization) to meet the demands for further physical and chemical tests as well as tissue models and animal tests. Directed evolution of NisB and NisC enzymes for hard-to-modify substrates is ongoing.

Some possibilities are the engineering of Ala1 instead of Gly1 and the replacement of Ser2 by Thr2 in the vasopressin sequence to assess whether the modifications will be more efficient and the yields improved. Hydrophobic, non-aromatic amino acids are also considered to be direct flanking of dehydratable serine or threonine to enhance the dehydratase activity (Rink et al., 2005). Moreover, although it is not essential for the activities of vasopressin, the C-terminal amidoglycine could be chemically installed after purification (Zou et al., 2002). Alternatively, a completely different enzymatic machinery could be employed, such as the published ProcM (Mukherjee and van der Donk, 2014) or the yet unpublished SyncM (Arias Orozco and Kuipers, manuscript in preparation). A next study will address these possibilities to increase the yield of fully modified vasopressin with a thioether bond.

## Author Contributions

OPK conceived the study. QL, MM-L, and OPK planned, conducted, and analyzed the experiments, drafted the manuscript, and interpreted the data. QL conducted the experiments. All authors read, critically revised, and approved the final manuscript.

## Conflict of Interest Statement

The authors declare that the research was conducted in the absence of any commercial or financial relationships that could be construed as a potential conflict of interest.
