# Supplementary material for: Feasability of Introducing a Thioether Ring in Vasopressin by nisBTC Co-expression in Lactococcus lactis
Source: Front Microbiol. 2019 Jul 2;10:1508. doi: 10.3389/fmicb.2019.01508 (PMC6614560; doi:10.3389/fmicb.2019.01508)
Supplement: Supplementary file 1 [file Table_1.DOCX]

**Supplementary Materials**

Supplementary Table 1 Primers for PCRs and sequencing used in this study

| No. | Products | Template | Primer | Nucleotide Sequences |
| --- | --- | --- | --- | --- |
| 1 | pNZE-nisleader-G-VSP | pNZnisA-E3 | G-VSP-fwd | GGTAGTTATTTCCAAAATTGTCCACGAGGATAAGCTTTCT TTGAACCAAAATTAG |
|  |  |  | leader-G-VSP-rev | TTTTGGAAATAACTACCGCGTGGTGATGCACCTGAATC |
| 2 | pNZE-nisleader-ASPRG-VSP | pNZnisA-E3 | ASPR-G-VSP-fwd | GCTAGTCCAAGAGGTAGTTATTTCCAAAATTGTCCACGAGGATAAGCTTTCTTTGAACCAAAATTAG |
|  |  |  | leader- ASPR-G-VSP-rev | ACTACCTCTTGGACTAGCGCGTGGTGATGCACCTGAATC |
| 3 | pNZE-nisleader-NG-VSP | pNZnisA-E3 | NG-VSP-fwd | AATGGTAGTTATTTCCAAAATTGTCCACGAGGATAAGCTTTCTTTGAACCAAAATTAG |
|  |  |  | leader-NG-VSP-rev | TTGGAAATAACTACCATTGCGTGGTGATGCACCTGAATC |
| 4 | pNZE-nisleader-MG-VSP | pNZnisA-E3 | MG-VSP-fwd | ATGGGTAGTTATTTCCAAAATTGTCCACGAGGATAAGCTTTCTTTGAACCAAAATTAG |
|  |  |  | leader-MG-VSP-rev | TTGGAAATAACTACCCATGCGTGGTGATGCACCTGAATC |
| 5 | pNZE-nisleader-WG-VSP | pNZnisA-E3 | WG-VSP-fwd | TGGGGTAGTTATTTCCAAAATTGTCCACGAGGATAAGCTTTCTTTGAACCAAAATTAG |
|  |  |  | leader-WG-VSP-rev | TTGGAAATAACTACCCCAGCGTGGTGATGCACCTGAATC |
| 6 | pNZE-nisleader-EG-VSP | pNZnisA-E3 | EG-VSP-fwd | GAAGGTAGTTATTTCCAAAATTGTCCACGAGGATAAGCTT TCTTTGAACCAAAATTAG |
|  |  |  | leader-EG-VSP-rev | TTGGAAATAACTACCTTCGCGTGGTGATGCACCTGAATC |
| 7 | pNZE-nis(Δ23-34)-G-VSP | pNZnisA-E3 | G-VSP-fwd | GGTAGTTATTTCCAAAATTGTCCACGAGGATAAGCTTTCT TTGAACCAAAATTAG |
|  |  |  | (Δ23-34)-G-VSP-rev | TTTTGGAAATAACTACCTTTCATGTTACAACCCATCAG |
| 8 | pNZE-nis(Δ23-34)-ASPRG-VSP | pNZnisA-E3 | ASPR-G-VSP-fwd | GCTAGTCCAAGAGGTAGTTATTTCCAAAATTGTCCACGAGGATAAGCTTTCTTTGAACCAAAATTAG |
|  |  |  | (Δ23-34)-ASPRG-VSP - rev | ACTACCTCTTGGACTAGCTTTCATGTTACAACCCATCAG |
| 9 | pNZE- nis(Δ23-34)-NG-VSP | pNZnisA-E3 | NG-VSP-fwd | AATGGTAGTTATTTCCAAAATTGTCCACGAGGATAAGCTTTCTTTGAACCAAAATTAG |
|  |  |  | (Δ23-34)-NG-VSP-rev | TGGAAATAACTACCATTTTTCATGTTACAACCCATCAG |
| 10 | pNZE- nis(Δ23-34)-MG-VSP | pNZnisA-E3 | MG-VSP-fwd | ATGGGTAGTTATTTCCAAAATTGTCCACGAGGATAAGCTTTCTTTGAACCAAAATTAG |
|  |  |  | (Δ23-34)-MG-VSP-rev | GGAAATAACTACCCATTTTCATGTTACAACCCATCAGAG |
| 11 | pNZE- nis(Δ23-34)-WG-VSP | pNZnisA-E3 | WG-VSP-fwd | TGGGGTAGTTATTTCCAAAATTGTCCACGAGGATAAGCTTTCTTTGAACCAAAATTAG |
|  |  |  | (Δ23-34)-WG-VSP-rev | GGAAATAACTACCCCATTTCATGTTACAACCCATCAGAG |
| 12 | pNZE- nis(Δ23-34)-EG-VSP | pNZnisA-E3 | EG-VSP-fwd | GAAGGTAGTTATTTCCAAAATTGTCCACGAGGATAAGCTT TCTTTGAACCAAAATTAG |
|  |  |  | (Δ23-34)-EG-VSP-rev | GGAAATAACTACCTTCTTTCATGTTACAACCCATCAGAG |
| 13 | pNZ- nis(Δ23-34)-NG-VSP | pNZnisA leader his2 | NG-VSP-fwd | AATGGTAGTTATTTCCAAAATTGTCCACGAGGATAAGCTTTCTTTGAACCAAAATTAG |
|  |  |  | (Δ23-34)-NG-VSP-rev | TGGAAATAACTACCATTTTTCATGTTACAACCCATCAG |
| 14 | pNZ- nis(Δ23-34)-MG-VSP | pNZnisA leader his2 | MG-VSP-fwd | ATGGGTAGTTATTTCCAAAATTGTCCACGAGGATAAGCTTTCTTTGAACCAAAATTAG |
|  |  |  | (Δ23-34)-MG-VSP-rev | GGAAATAACTACCCATTTTCATGTTACAACCCATCAGAG |
| 15 | pNZ- nisA-NG-VSP | pNZnisA leader his2 | nisA-VSP-fwd | TCATACTTCCAAAACTGTCCACGTGGTTAAGCTTTCTTTG AACCAAAATTAG |
|  |  |  | nisA-NG-VSP rev | GTGGACAGTTTTGGAAGTATGAACCATTTTTGCTTACGTG AATACTACAATGACAAGT |
| 16 | pNZ- nisA-MG-VSP | pNZnisA leader his2 | nisA-VSP-fwd | TCATACTTCCAAAACTGTCCACGTGGTTAAGCTTTCTTTG AACCAAAATTAG |
|  |  |  | nisA-MG-VSP rev | GTGGACAGTTTTGGAAGTATGAACCCATTTTGCTTACGTG AATACTACAATGACAAGT |
| 17 |  |  | pNZE3Emf | CAATTCCTTAAAACATGCAGG |
| 18 |  |  | pNZcmfw | TTCAGGAATTGTCAGATAGG |

Primers 1-16 were used for PCR, primer 17 and primer 18 were used for sequencing.

Supplementary Figure 1 Two plasmids platform for biosynthesis and regulation of expression of nisin


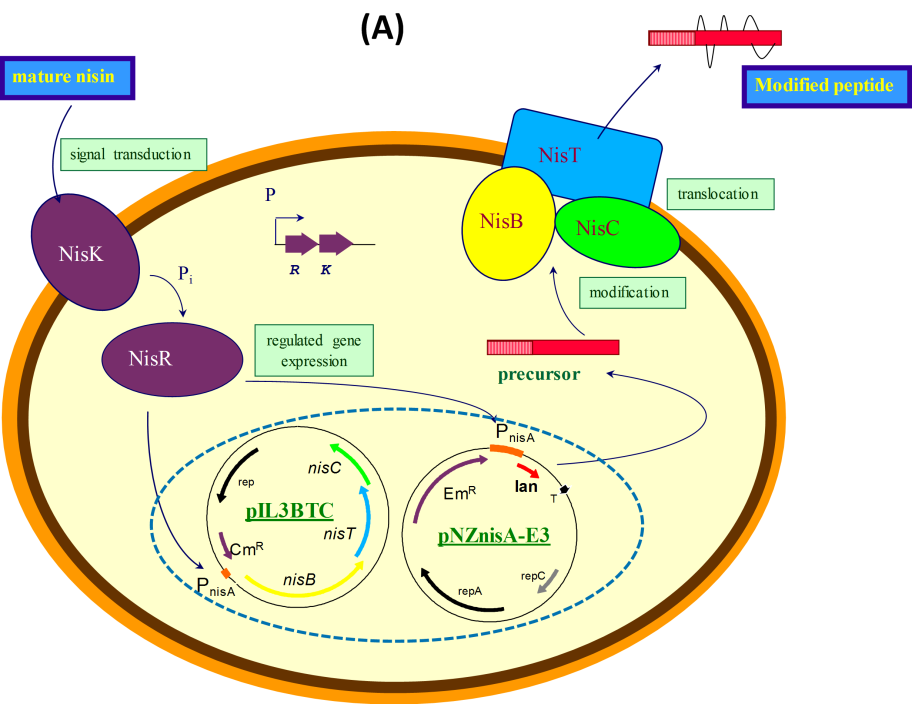

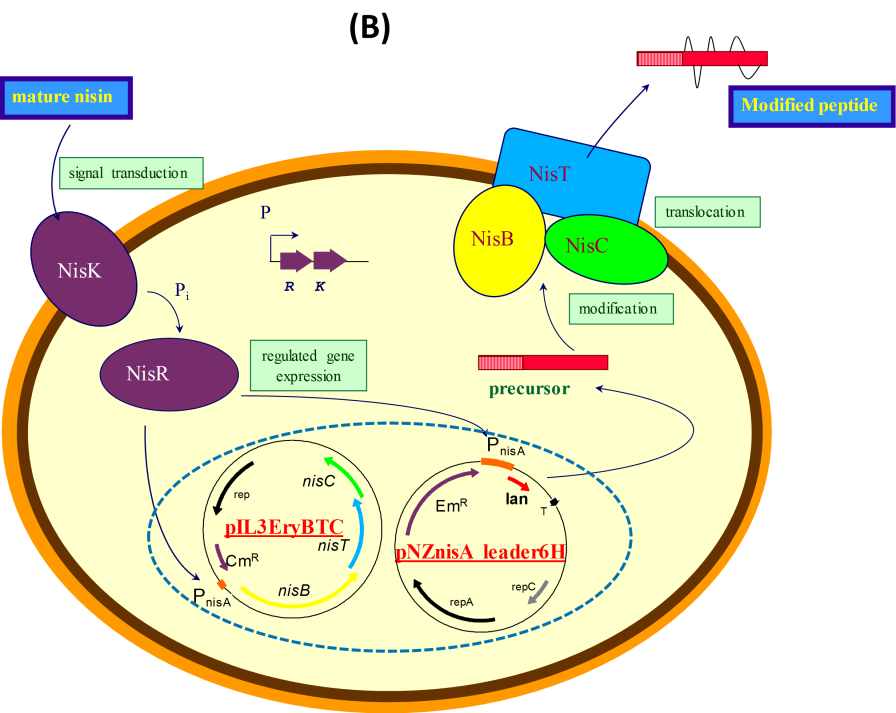


Adapted from and Oscar.p.kuipers *et al*, 1993, de Ruyter *et al*. 1996 and Lubelski J, *et al*, 2008.

The strain NZ9000 is a *L. lactis* strain derived from MG1363 which harbors the two-component system *nisRK* thus enabling an inducible gene expression system. (A) There are two plasmids in the expression strain *L. lactis* NZ9000 (pIL3BTC, pNZnisA-E3): pIL3BTC (with chloramphenicol resistance) and another plasmid “pNZnisA-E3” (with erythromycin resistance) encodes pre-nisin, therefore being responsible for expression. pIL3BTC encodes the dehydratase Nis B, cyclase NisC and transporter NisT. These three enzymes correspond to the post-translational modification machinery required. The two plasmids have been circled by blue dash.

(B) Two plasmids in the system are pIL3EryBTC (with erythromycin resistance) and pNZnisA-leader6H (with chloramphenicol resistance). The two plasmids have been circled by blue dash.

Supplementary Figure 2 MS results of peptides before and after digestion. Peptide MS spectra before (left) and after (right) digestion. The peptide sequences consistent with the measured mass are depicted.


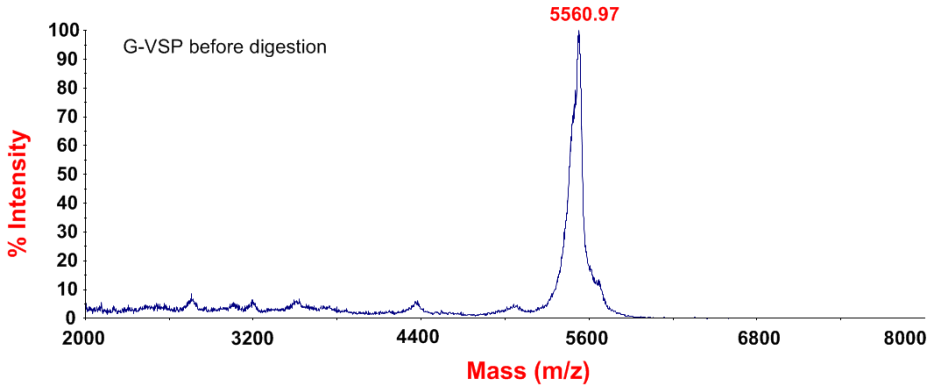

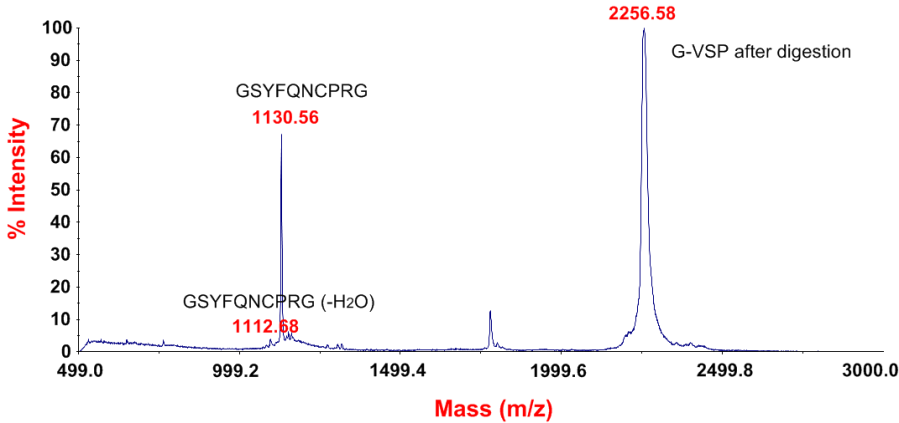


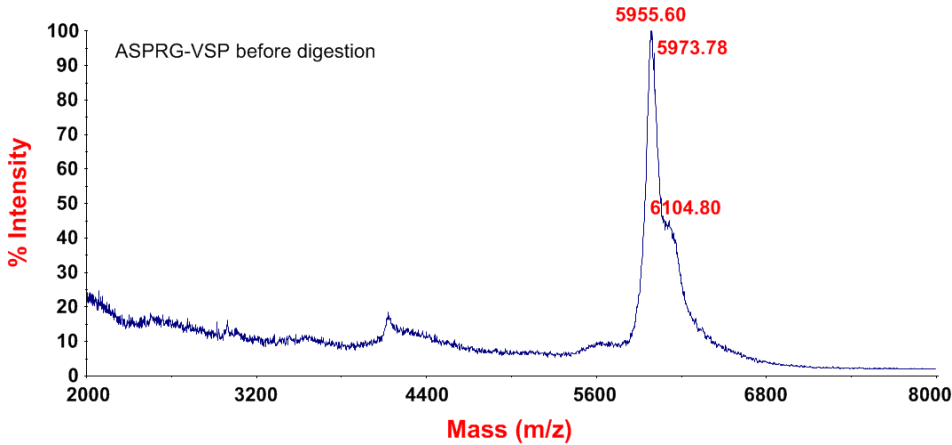

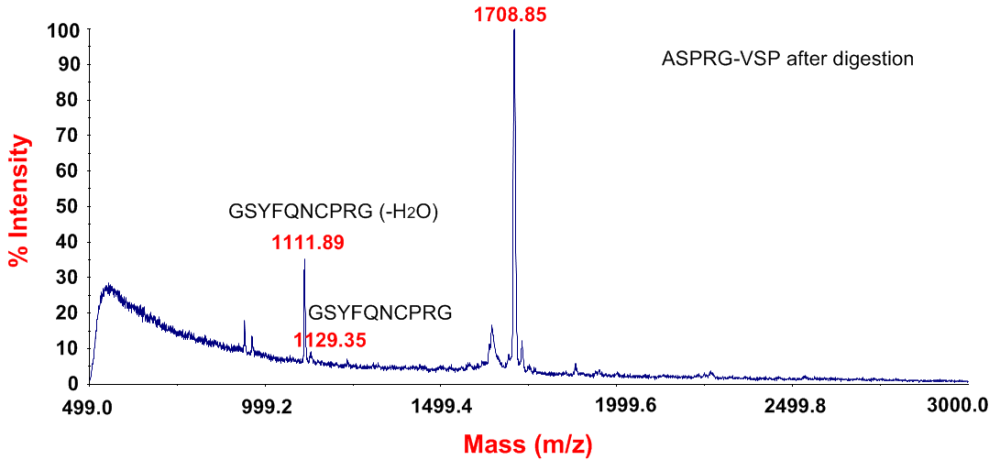


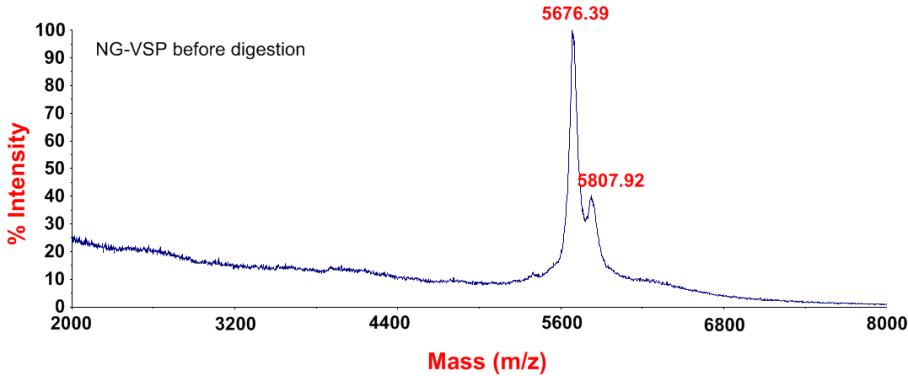

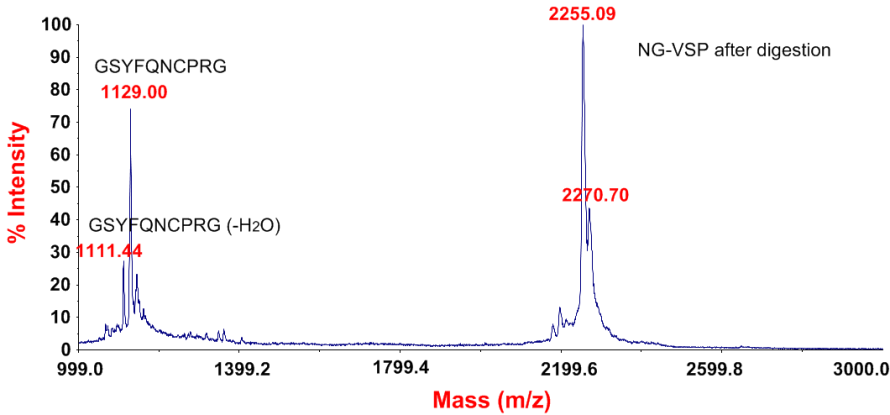


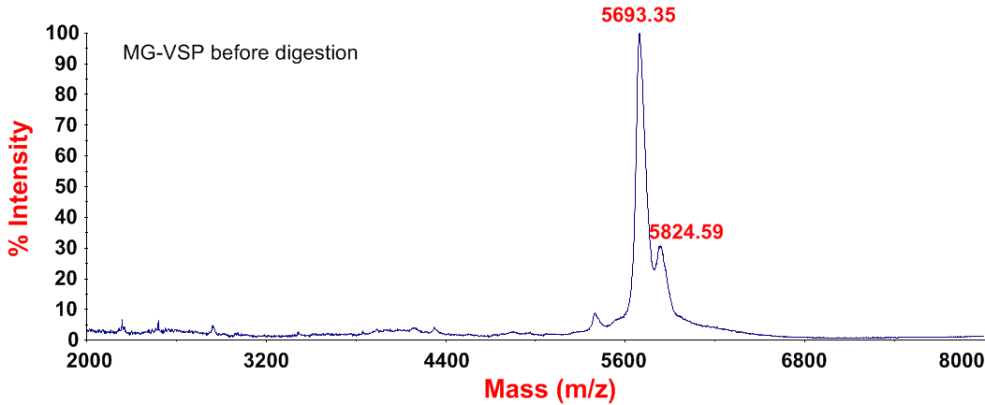

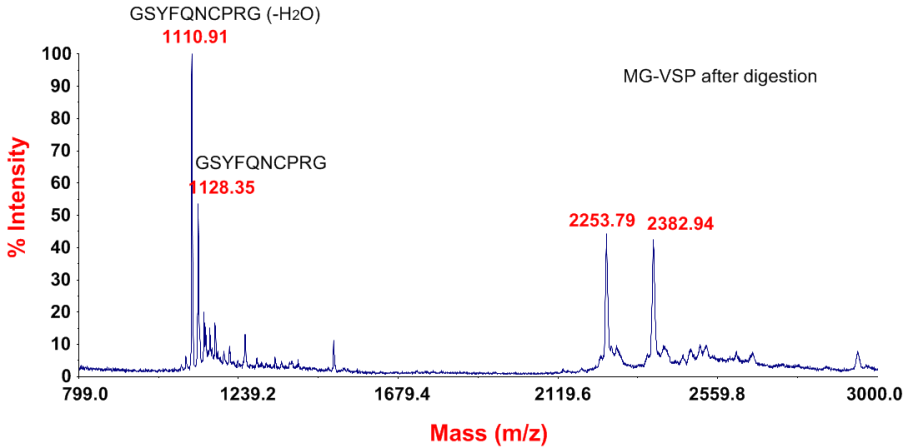


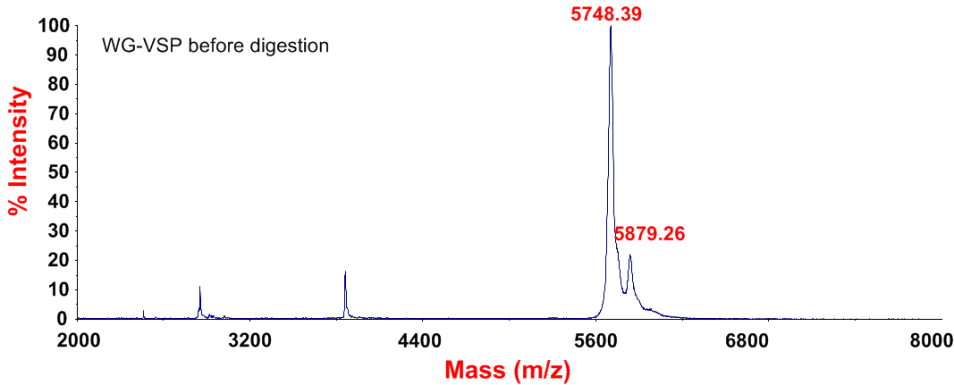

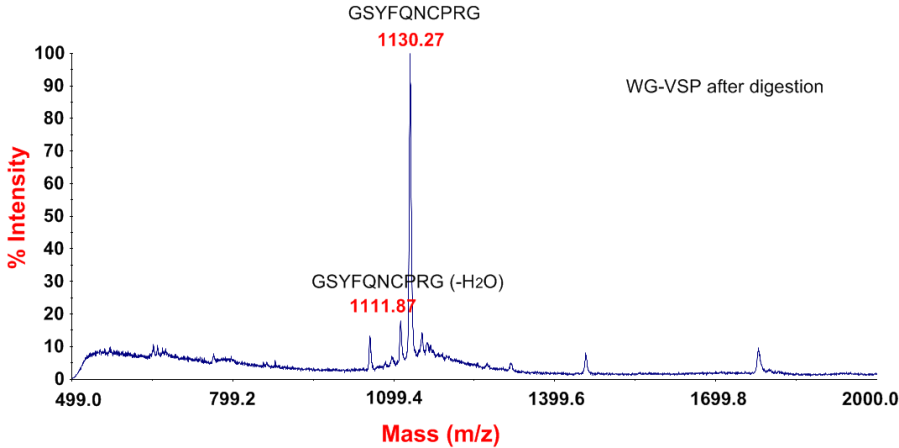


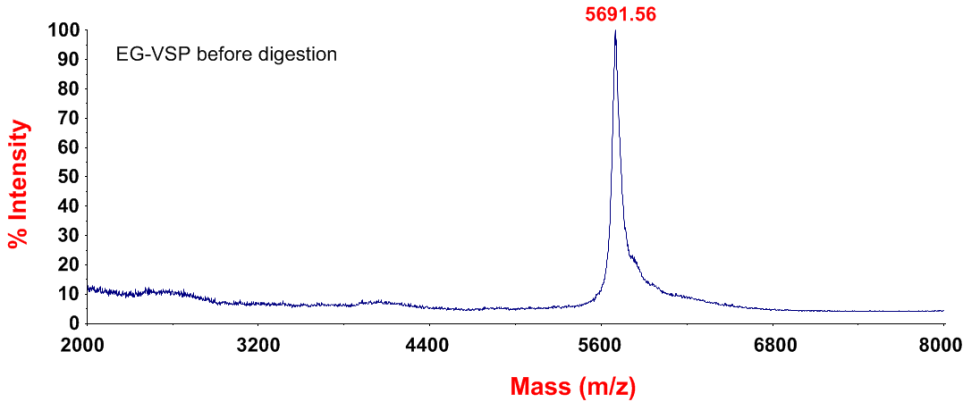

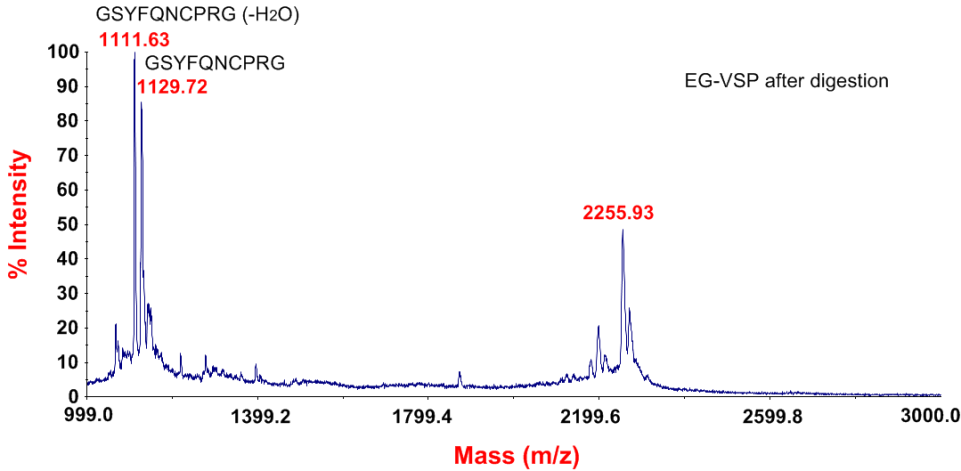


Supplementary Figure 3 LC-MS/MS results of peptides


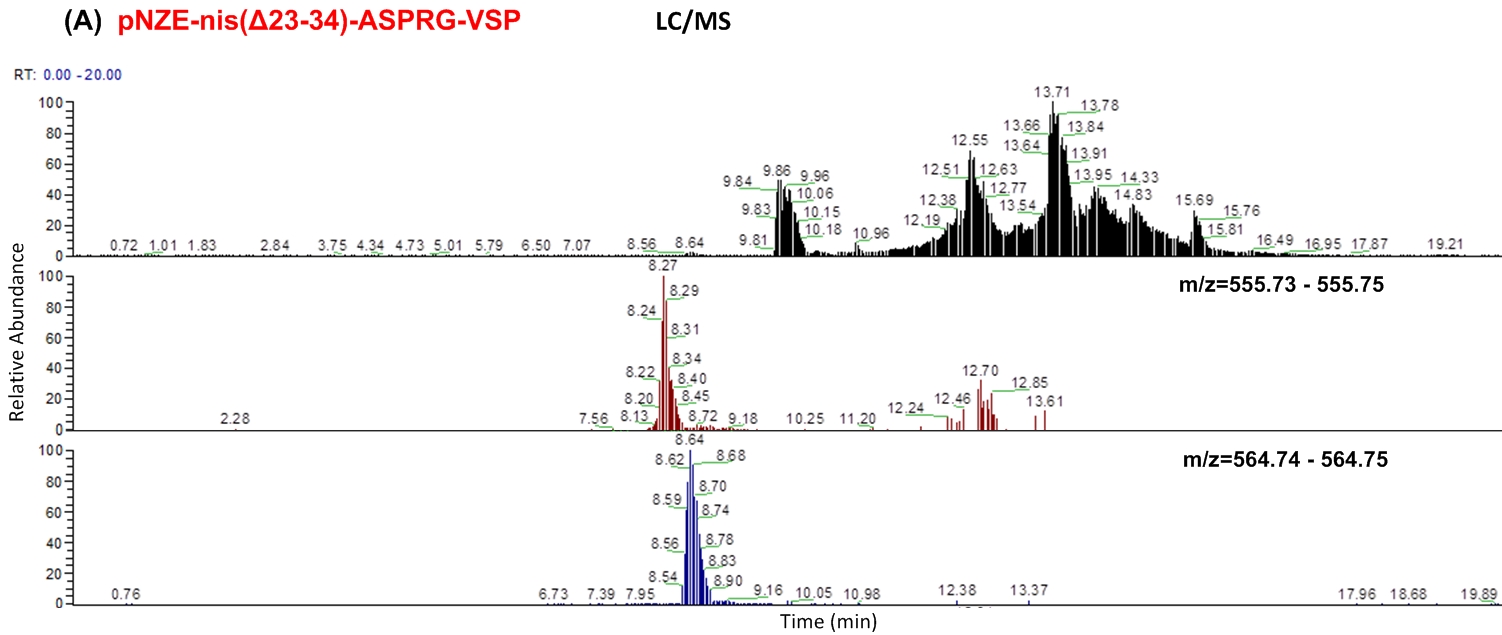


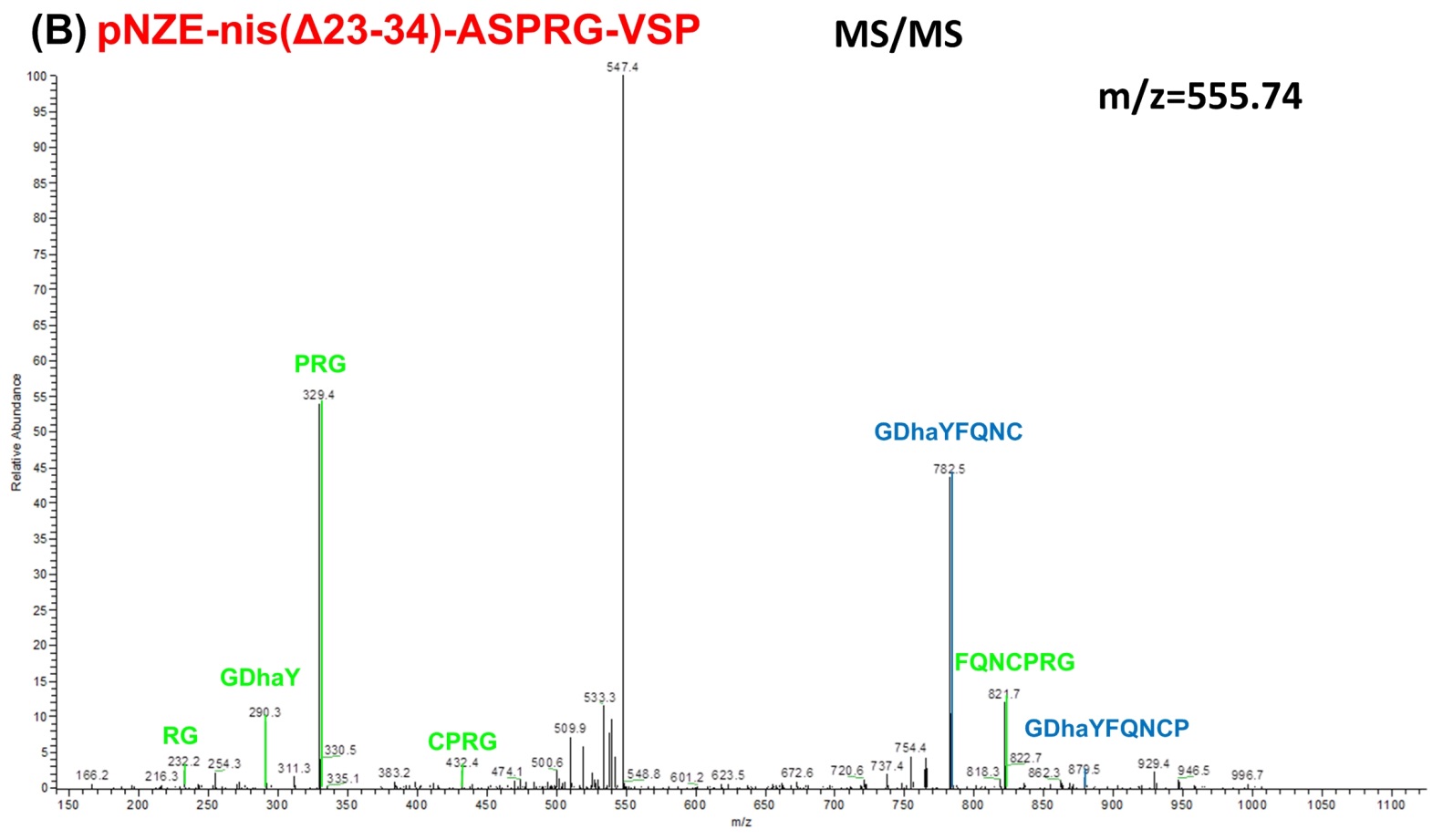


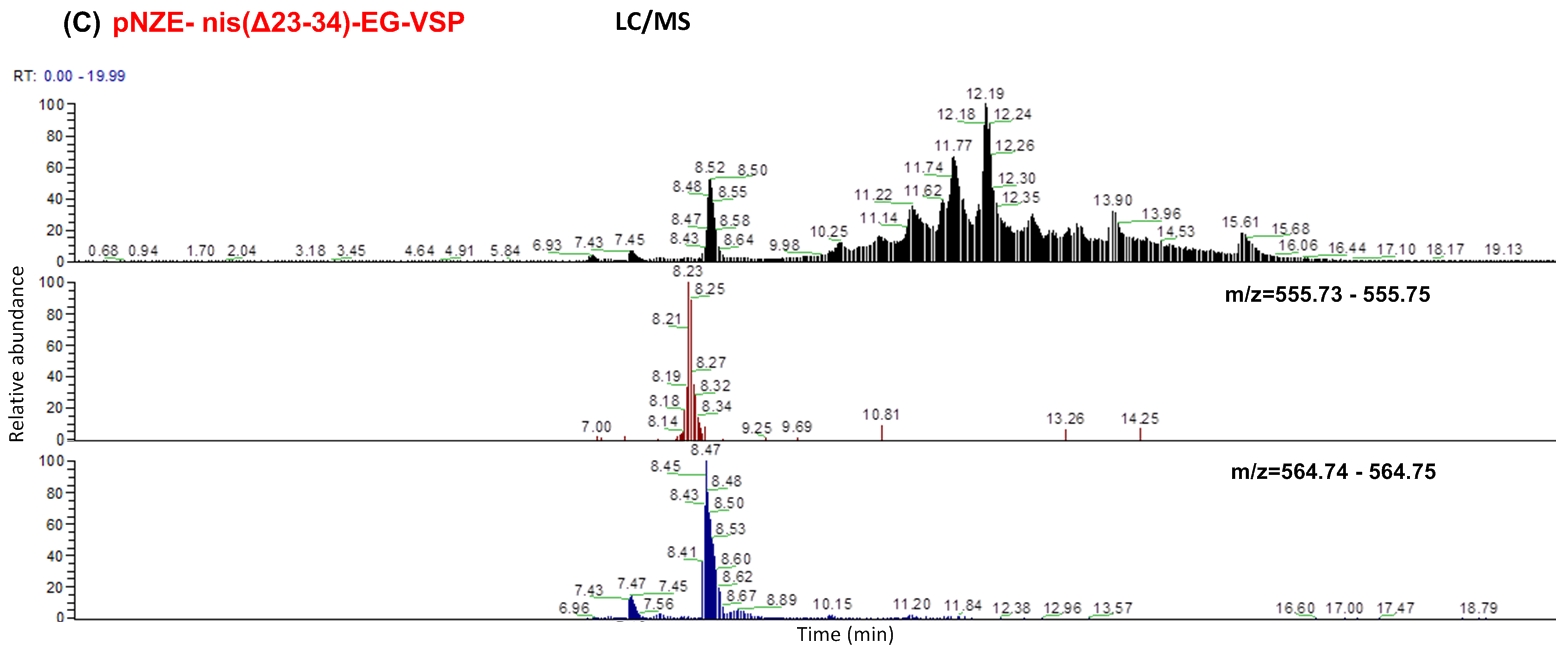


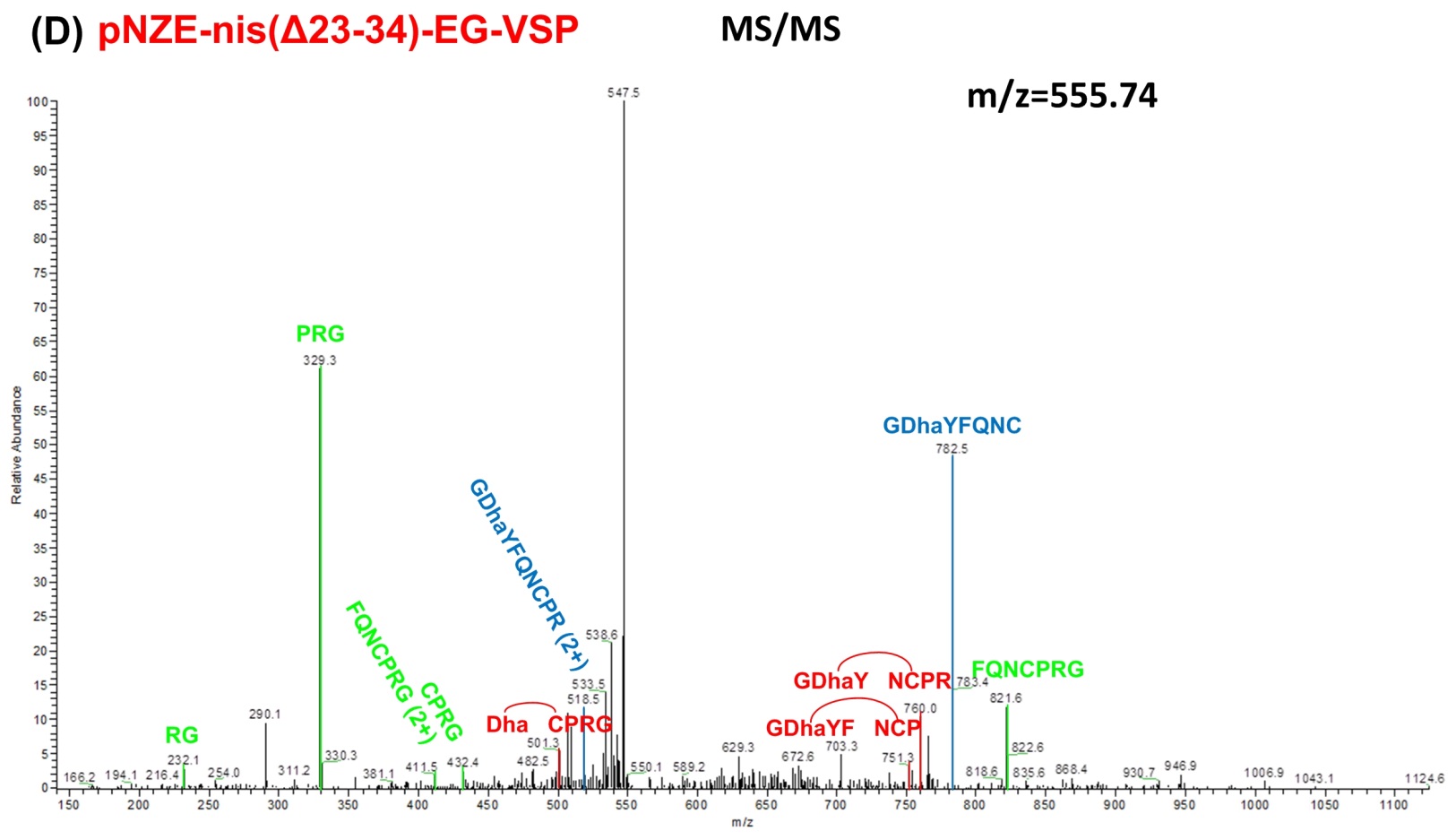


LC-MS/MS results of pNZE-nis(Δ23-34)-ASPRG-VSP ((A) and (B)) and pNZE-nis(Δ23-34)-EG-VSP ((C) and (D)) after digestion.

(A), (C) LC/MS results, m/z=555.73-555.75 correspond to the peptide with dehydrated serine (Dha), either linear or circled, m/z=564.74-564.75 correspond to the peptide without dehydration.

(B), (D) MS/MS results, m/z=555.74 correspond to the peptide with dehydrated serine (Dha), either linear or circled.

(B), MS/MS fragments of 7 characteristic fragments labelled as green (correspond to linear vasopressin) or blue (correspond to the fragment can be either linear vasopressin or cyclized vasopressin); (D), MS/MS fragments of 11 characteristic fragments labelled as green (correspond to linear vasopressin), red (cyclized vasopressin) or blue (correspond to the fragment can be either linear vasopressin or cyclized vasopressin); Dha, dehydroalanine; red line between Dha and cysteine indicates a thioether bridge.

peaks consistent with three specific fragments (DhaCPRG, GDhaYFNCP and GDhaYNCPR ) were detected in the vasopressin part of pNZE-nis(Δ23-34)-EG-VSP. It showed the formation of thioether ring.

References:

[1] O.P. Kuipers, M.M. Beerthuyzen, R.J. Siezen, and W.M. De Vos, Characterization of the nisin gene cluster *nisABTCIPR* of *Lactococcus lactis*: requirement of expression of the *nisA* and *nisI* genes for development of immunity. European journal of biochemistry 216 (1993) 281-291.

[2] P. De Ruyter, O.P. Kuipers, and W.M. De Vos, Controlled gene expression systems for *Lactococcus lactis* with the food-grade inducer nisin. Applied and environmental microbiology 62 (1996) 3662-3667.

[3] J. Lubelski, R. Rink, R. Khusainov, G.N. Moll, and O.P. Kuipers, Biosynthesis, immunity, regulation, mode of action and engineering of the model lantibiotic nisin. Cellular and molecular life sciences 65 (2008) 455-76.
